# Supplementary material for: A Humanized Yeast Phenomic Model of Deoxycytidine Kinase to Predict Genetic Buffering of Nucleoside Analog Cytotoxicity
Source: Genes (Basel). 2019 Sep 30;10(10):770. doi: 10.3390/genes10100770 (PMC6826991; doi:10.3390/genes10100770)
Supplement: Supplementary file 1 [file genes-10-00770-s001.zip › Additional_Files/Additional_File7_GOTermHeatmaps.zip!/Additional_File7_GOTermHeatmaps/DNA-directed_RNA_polymerase_II,_holoenzyme.pdf]

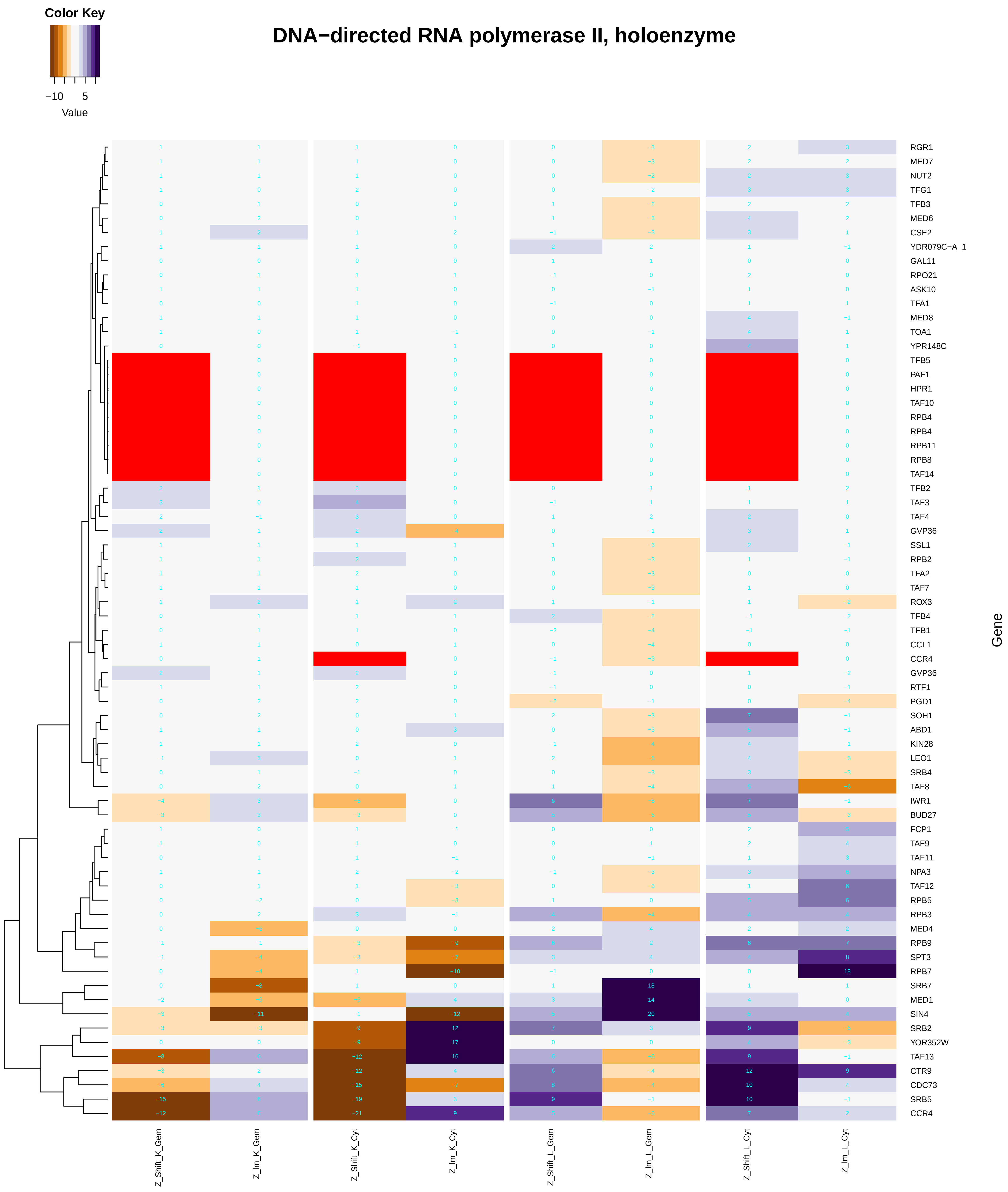
DNA−directed RNA polymerase II, holoenzyme
Color Key
−10
5
Value
RGR1
MED7
NUT2
TFG1
TFB3
MED6
CSE2
YDR079C−A_1
GAL11
RPO21
ASK10
TFA1
MED8
TOA1
YPR148C
TFB5
PAF1
HPR1
TAF10
RPB4
RPB4
RPB11
RPB8
TAF14
TFB2
TAF3
TAF4
GVP36
SSL1
RPB2
TFA2
TAF7
ROX3
TFB4
TFB1
CCL1
CCR4
GVP36
RTF1
PGD1
SOH1
ABD1
KIN28
LEO1
SRB4
TAF8
IWR1
BUD27
FCP1
TAF9
TAF11
NPA3
TAF12
RPB5
RPB3
MED4
RPB9
SPT3
RPB7
SRB7
MED1
SIN4
SRB2
YOR352W
TAF13
CTR9
CDC73
SRB5
CCR4
Gene
Z_Shift_K_Gem
Z_Im_K_Gem
Z_Shift_K_Cyt
Z_Im_K_Cyt
Z_Shift_L_Gem
Z_Im_L_Gem
Z_Shift_L_Cyt
Z_Im_L_Cyt
